# Surgical valve in valve implantation in pregnancy… a dilemma in the management of mechanical valves

**DOI:** 10.1186/1749-8090-8-S1-O69

**Published:** 2013-09-11

**Authors:** M Krishnan, I Manoly, D Karunaratne, A Hoschtitzky, R Hasan

**Affiliations:** 1 Cardiac Surgery, Manchester Heart Centre, Central Manchester University Hospitals NHS Foundation Trust, Manchester, UK; 2 Manchester Heart Centre, Central Manchester University Hospitals NHS Foundation Trust, Manchester, UK

## Introduction

The choice of valve and anticoagulation for young female patients of childbearing age has always been a major topic of discussion, posing a challenge in the management of this cohort of patients. We describe a successful case of a redo-aortic valve replacement for a young pregnant female who presented with severe aortic valve incompetence due to valve thrombosis in her third trimester, which is the first reported case of surgical valve in valve implantation to be described.

## Case report

A 25-year-old pregnant female with complex cardiac history presented with severe shortness of breath. She was known to have congenital aortic valve stenosis. She underwent double root replacement with mechanical aortic valve and tissue pulmonary valve prosthesis and was subsequently started on warfarin.

In 2011, she became pregnant again. Warfarin was hence stopped and therapeutic dose of heparin commenced and was closely monitored.. At 31 weeks of pregnancy, she presented with severe breathlessness and was diagnosed with severe aortic incompetence due to thrombosis of her mechanical aortic valve. After discussion, she underwent C-section under full back up followed by redosternotomy 2 days later. As a result of her previous cardiac surgeries, the valve was completely embedded, an attempt to repair it was futile. The leaflets of the original valve were removed and a mechanical valve was seated on top of the previous valve. There was no major post-operative complications to her or her baby and were discharged home.

## Discussion

Currently, there are no guidelines on how to manage pregnant patient with thrombosed mechanical valve. Few recommend C-section followed by valve replacement under CPB to prevent maternal mortality. There is a risk of severe postpartum haemorrhage and fetal mortality with CPB. It clearly demonstrated the interdependency of various specialities in tackling such complex issues and we hope to have the guidelines set in the same manner.

